# Histone methyltransferase SETD2 inhibits tumor growth via suppressing CXCL1-mediated activation of cell cycle in lung adenocarcinoma

**DOI:** 10.18632/aging.104120

**Published:** 2020-11-20

**Authors:** You Zhou, Xiao Zheng, Bin Xu, Haifeng Deng, Lujun Chen, Jingting Jiang

**Affiliations:** 1 Department of Tumor Biological Treatment, The Third Affiliated Hospital of Soochow University, Changzhou 213003, China; 2 Jiangsu Engineering Research Center for Tumor Immunotherapy, Changzhou 213003, China; 3 Institute of Cell Therapy, Soochow University, Changzhou 213003, China

**Keywords:** lung adenocarcinoma, SETD2, CXCL1, cell cycle

## Abstract

The histone H3 lysine 36 methyltransferase SET-domain-containing 2 (SETD2) has been reported to be frequently mutated or deleted in many types of human cancer. However, the role of SETD2 in lung adenocarcinoma (LUAD) has not been well documented. In the present study, we found that SETD2 was significantly down-regulated both in LUAD tissues and cell lines. Functionally, the increased expression of SETD2 significantly attenuated the proliferation of cancer cells by affecting the cell cycle, whereas SETD2 deficiency dramatically improved these proliferative abilities of cancer cells. Through conjoint analysis of RNA-seq and ChIP data, we identified a functional target gene of SETD2, CXCL1, and its expression was negatively correlated with that of SETD2. Moreover, SETD2 deletion stimulated cell cycle-related proteins to promote LUAD. Further mechanistic studies demonstrated that histone H3 lysine 36 trimethylation (H3K36me3) catalyzed by SETD2 interacted with the promoter of CXCL1 to regulate its transcription and downstream signaling pathways, contributing to tumorigenesis *in vitro* and *in vivo*. Our findings suggested that SETD2 inhibited tumor growth *via* suppressing CXCL1-mediated activation of cell cycle, indicating that the regulation of H3K36me3 level by targeting SETD2 and/or the administration of downstream CXCL1 might represent a potential therapeutic way for new treatment in LUAD.

## INTRODUCTION

Epigenetic regulators play important roles in tumor evolution, among which histone methyltransferases (HMTs) have increasingly become the appealing therapeutic targets for cancer disease interventions because they are frequently dysregulated in a spectrum of human cancers [1]. Differentially deposited histone methylation modifications mediated by distinct HMTs can govern gene transcription. Genome-wide studies show that H3K36me3 mostly distributes in the gene body in a 3′ end enriched manner like the Ser2 phosphorylated RNAPII and maintains the repressive chromatin status. In addition, H3K36me3 acts as a safeguard to prevent aberrant transcriptional initiation from cryptic gene promoters [2–5]. SET-domain-containing 2 (SETD2) is the major HMT catalyzing the H3K36me3 [6]. Previous studies have suggested that SETD2-silenced cells exhibit deficiency in chromosome segregation and DNA repair [7–9]. SETD2-catalyzed H3K36me3 has been shown to recruit DNMT3b to ensure the fidelity of gene transcription initiation in embryonic cells [10]. Moreover, H3K36me3 mediated by SETD2 has been implicated in RNA splicing during gene transcription, and it affects the alternative splicing of a subset of genes involved in tumorigenesis [11–13]. In accordance with the crucial roles of SETD2 in maintaining chromosome stabilization and integrity and regulating gene transcription, SETD2 has been reported to be frequently mutated or deleted in many types of human cancer [14–17].

Lung adenocarcinoma (LUAD) is a major subtype of non-small cell lung cancer (NSCLC), which is the leading cause of cancer-related death worldwide. However, the genetic characterization and molecular mechanisms of SETD2 in tumorigenesis of LUAD remain largely undetermined.

In the present study, we showed that SETD2 functioned as a putative tumor suppressor in LUAD using human LUAD tissue specimens and cell lines in combination with gene expression profile obtained from The Cancer Genome Atlas (TCGA). We found that elevated levels of SETD2 significantly attenuated the proliferation of cancer cells by affecting cell cycle, whereas SETD2 deficiency dramatically improved these proliferative abilities of cancer cells. Notably, chemokine (C-X-C motif) ligand 1 (CXCL1) was identified as one functional downstream gene of SETD2. The mechanistic investigation demonstrated that H3K36me3 catalyzed by SETD2 interacted with the promoter of CXCL1 to regulate its transcription and downstream signaling pathways, contributing to tumorigenesis *in vitro* and *in vivo*. In addition, SETD2 deletion up-regulated the expression of CXCL1 and stimulated cell cycle-related proteins to promote LUAD. Collectively, our study revealed an in facto regulatory mechanism by which SETD2 deficiency determined the CXCL1-mediated activation of cell cycle and consequent tumorigenesis.

## RESULTS

### SETD2 expression is down-regulated in human LUAD

**Figure 1:**
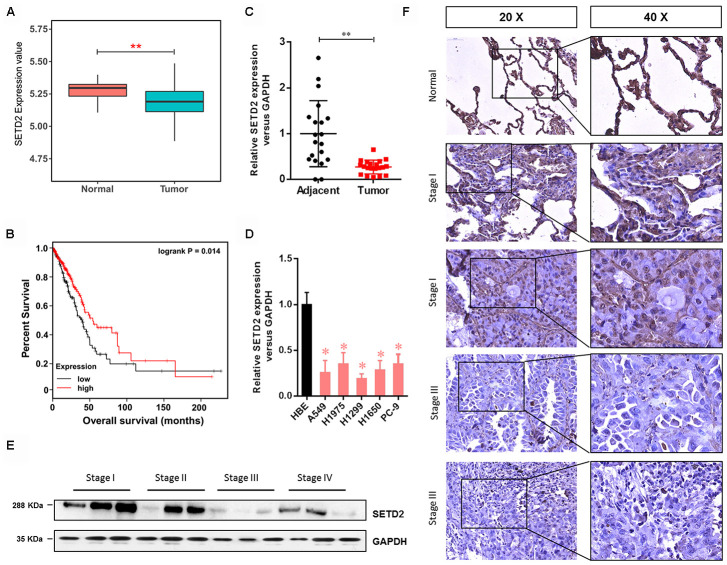
**SETD2 is clinically relevant in human LUAD.** (**A**) Box plot of SETD2 expression levels in patients with lung cancer from TCGA. (**B**) The association of SETD2 expression at the mRNA level and overall survival using TCGA data (*P* values by log-rank test). (**C**) Scatter plot of SETD2 expression levels in LUAD tumors and adjacent normal epithelial tissues. (**D**) Real-time qPCR analysis of SETD2 expression in HBE cells and human lung cancer cell lines A549, H1975, H1299, H1650 and PC-9. (**E**) Western blotting analyses of SETD2 expression at the protein level in four clinical stages of lung cancer progression using human lung tissue specimens. (**F**) SETD2 staining of human lung cancer tissues with four clinical stages of cancer progression. Scale bars: 50 μm. *P<0.05, **P<0.01. Two-tailed Student’s *t* test.

To explore the possible roles of SETD2 in LUAD, we first examined the gene expression profile from TCGA, which indicated that the SETD2 expression was significantly decreased in tumors compared with normal counterparts (Figure 1A). The Kaplan-Meier analyses showed that low expression of SETD2 was significantly associated with a poor prognosis in LUAD patients (Figure 1B), providing important prognostic information for risk stratification of overall survival. Simultaneously, the SETD2 expression pattern was also examined using human LUAD tissue specimens. Results showed that the expression level of SETD2 was significantly decreased in tumors compared with normal adjacent lung epithelial tissues (Figure 1C). Likewise, human lung cancer cell lines (A549, H1975, H1299, H1650 and PC-9) showed a significantly lower expression of SETD2 compared with HBE cell line (Figure 1D). In addition, western blotting analyses and IHC staining revealed that the SETD2 expression at the protein level was inversely correlated with the clinical stage of LUAD (Figures 1E, 1F). The correlation between SETD2 expression and patients’ clinical parameters in human LUAD tissues was summarized in Table 1. Our data demonstrated that SETD2 expression was significantly associated with tumor size (*P* = 0.012) and tumor stage (*P* = 0.027) of the patients. Taken together, these findings highlighted SETD2 as a prognostic biomarker for LUAD patients.

**Table 1:** Correlation between SETD2 expression in LUAD tissues and patients’ clinical parameters.

**Clinical Parameters**	**Cases**	**SETD2 expression level**		
		***High***	***Low***	***P***
Gender				0.653
Male	49	22	27	
Female	41	13	28	
Age (years)				0.472
<62	45	31	14	
≥62	45	25	20	
Tumor diameter (cm)				0.012*
≤5	69	11	58	
>5	21	6	15	
Lymphatic metastasis				0.245
Yes	6	1	5	
No	84	35	49	
TNM stage				0.027*
*I+II*	65	28	37	
*III+IV*	25	4	21	

* Chi-squared test. *P<0.05

### Overexpression of wildtype SETD2 inhibits cancer cell growth *in vitro*

**Figure 2:**
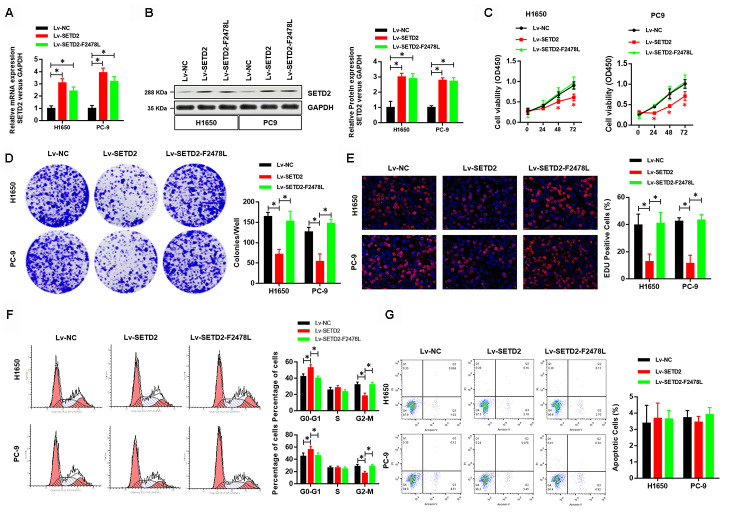
**Overexpression of wildtype SETD2 inhibits cancer cell growth *in vitro*.** (**A**) Real-time qPCR showed the expression level of wildtype or catalytically dead version of SETD2 (F2478L mutant) in lung cancer cells H1650 and PC-9 and its overexpression. (**B**) Western blotting analyses of wildtype or catalytically dead version of SETD2 (F2478L mutant) overexpression in H1650 and PC-9 cells. Quantitative results were shown in the right panel. (**C**) Cell proliferation assays of wildtype or catalytically dead version of SETD2 (F2478L mutant) overexpressing H1650 and PC-9 cells. (**D**) Anchorage-independent growth assays of wildtype or catalytically dead version of SETD2 (F2478L mutant) overexpressing H1650 and PC-9 cells. Quantitative results were indicated in the right panel. (**E**) EDU staining of wildtype or catalytically dead version of SETD2 (F2478L mutant) overexpressed H1650 and PC-9 cells (×200). (**F**) Cell cycle analysis of wildtype or catalytically dead version of SETD2 (F2478L mutant) overexpressing H1650 and PC-9 cells. Proportion of cells in G0-G1 phase, S phase and G2-M phase was quantified. (**G**) Apoptosis analysis of wildtype or catalytically dead version of SETD2 (F2478L mutant) overexpressed H1650 and PC-9 cells by Annexin V/PI assay. *P<0.05, **P<0.01. Two-tailed Student’s *t* test.

To investigate the effect of SETD2 on phenotypes of lung cancer cells *in vitro*, we first ectopically overexpressed a wildtype or a catalytically dead version of SETD2 (F2478L mutant) in lung cancer cells H1650 and PC-9. Real-time qPCR and western blotting analyses were performed to confirm the overexpression of SETD2 (Figures 2A, 2B). Cell viability analysis revealed that increased expression of wildtype SETD2 significantly attenuated the proliferation of cancer cells (Figure 2C). Moreover, colony formation (Figure 2D) and EDU assay (Figure 2E) were markedly inhibited upon wildtype SETD2 overexpression. Next, we examined whether SETD2 affected cell cycle phase. Flow cytometry was used for cell cycle phase analyses in H1650 and PC-9 cells. Results demonstrated that the proportions of cells in G0-G1 phase and G2-M phase were significantly increased and decreased upon wildtype SETD2 overexpression, respectively, while the proportion of cells in S phase remained unchanged, suggesting a S phase arrest in wildtype SETD2-overexpressing lung cancer cells (Figure 2F). However, the overexpression of wildtype or catalytically dead version of SETD2 (F2478L mutant) in lung cancer cells H1650 and PC-9 showed no effect on the apoptosis level as measured by Annexin V/PI assay (Figure 2G). Notably, the overexpression of catalytically dead version of SETD2 showed none effect on these parameters of H1650 and PC-9 cells, which suggested that over-expression this kind of large protein showed no toxic to the H1650 and PC-9 cells.

**Figure 3:**
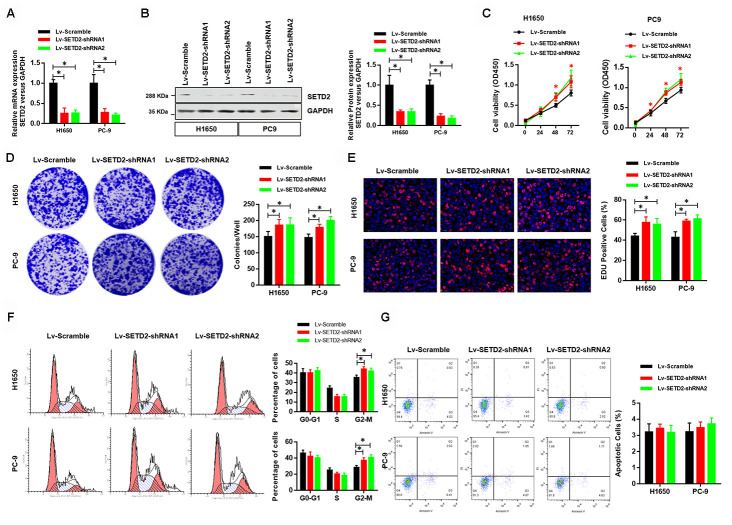
**Deficiency of SETD2 improves cancer cell growth *in vitro*.** (**A**) Real-time qPCR confirmed the downregulated expression level of SETD2 in lung cancer cells H1650 and PC-9. (**B**) Western blotting analyses of SETD2 deficiency in H1650 and PC-9 cells. Quantitative results were shown in the right panel. (**C**) Cell proliferation assays of SETD2 deficiency H1650 and PC-9 cells. (**D**) Anchorage-independent growth assays of SETD2 deficiency H1650 and PC-9 cells. Quantitative results were indicated in the right panel. (**E**) EDU staining of SETD2 down-regulated H1650 and PC-9 cells (×200). (**F**) Cell cycle analysis of SETD2 deficiency H1650 and PC-9 cells. Proportion of cells in G0-G1 phase, S phase and G2-M phase was quantified. (**G**) Apoptosis analysis of SETD2 down-regulated H1650 and PC-9 cells by Annexin V/PI assay. *P<0.05. Two-tailed Student’s *t* test.

To further illustrate the critical role of SETD2 on proliferation of lung cancer cells *in vitro*, we next knock-downed the level of SETD2 in lung cancer cells H1650 and PC-9 *via* two lentivirus-mediated shRNAs (shRNA1 and shRNA2) targeting SETD2. Real-time qPCR and western blotting analyses were performed to confirm the SETD2 deficiencies (Figures 3A, 3B). Deletion of SETD2 significantly improved the proliferation, colony formation and EDU positive cells (Figures 3C–3E) abilities of cancer cells. Also, SETD2 deficiency improved the G2-M phase and impaired the S phase (Figure 3F). Moreover, SETD2 deficiency did not affect the apoptosis of H1650 and PC-9 cells (Figure 3G). These results collectively implicated that SETD2 inhibited the cell proliferation and cell cycle of LUAD cells.

### SETD2 negatively regulates CXCL1 expression

**Figure 4:**
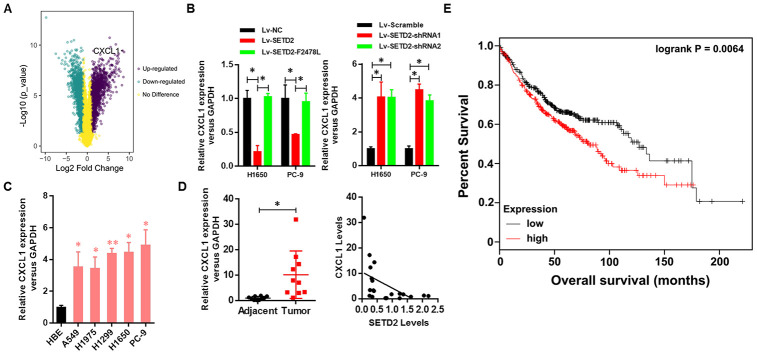
**Clinical relevance of CXCL1 in human LUAD.** (**A**) CXCL1 was up-regulated upon SETD2 depletion as indicated in volcano plot. (**B**) Real-time qPCR analysis of CXCL1 expression level upon SETD2 overexpression or depletion in H1650 and PC-9 cells. (**C**) Real-time qPCR analysis of CXCL1 expression in HBE cells and human lung cancer cell lines A549, H1975, H1299, H1650 and PC-9. (**D**) Scatter plot of CXCL1 expression levels in LUAD tumors and adjacent normal epithelial tissues (left panel). The association (by Pearson’s) between SETD2 and CXCL1 expressions in patients (right panel). (**E**) Kaplan-Meier plot of overall survival based on the CXCL1 expression index in patients (*P* values by log-rank test). *P<0.05. Two-tailed Student’s *t* test. Pearson’s correlation test was used to analysis the correlation between CXCL1 and SETD2.

As our above-mentioned data demonstrated that overexpression of SETD2 could suppress cancer cell growth *in vitro*, we hypothesized that some of SETD2- and/or H3K36me3-regualted genes would affect tumorigenesis. Here, we focused on up-regulated genes by SETD2 deficiency. First, SETD2-silenced H1650 cells were generated using three shRNA constructs, which displayed similar knockdown efficiency (Supplementary Figure 1). Then we performed RNA sequencing (RNA-seq) analysis using H1650 cells with or without SETD2 depletion to identify SETD2-regulated genes. Among 1,501 genes expressed, 990 genes were up-regulated, and 511 genes were down-regulated (fold change > 5) in SETD2-silenced cells (Supplementary Table 1). Notably, CXCL1 was found to be significantly up-regulated upon SETD2 depletion (Figure 4A and Supplementary Figure 2), acting as an appealing downstream target of SETD2.

To further investigate the negative regulation of SETD2 on CXCL1 in lung cancer, we examined the correlated expression between SETD2 and CXCL1. Results indicated that the CXCL1 expression was down-regulated by SETD2 overexpression in lung cancer cells H1650 and PC-9, while SETD2 deletion up-regulated the expression of CXCL1 in these two cell lines (Figure 4B). Meanwhile, the lung cancer cell lines (A549, H1975, H1299, H1650 and PC-9) showed a remarkably higher expression level of CXCL1 compared with HBE cell line (Figure 4C), showing an opposite expression pattern of SETD2 (Figure 1D).

Next, the clinical relevance of CXCL1 was assessed by analyses of human LUAD tissues and corresponding patient information. The expression of CXCL1 was elevated in tumors compared with adjacent normal lung tissues, whereas its expression was negatively correlated with SETD2 expression (Figure 4D). Kaplan-Meier analyses showed that CXCL1 overexpression was correlated with worse overall survival in patients with lung cancer (Figure 4E). Collectively, these results suggested that SETD2 negatively regulated CXCL1, which could function as a prognostic biomarker in LUAD.

### SETD2 depletion stimulates cell cycle progression

**Figure 5:**
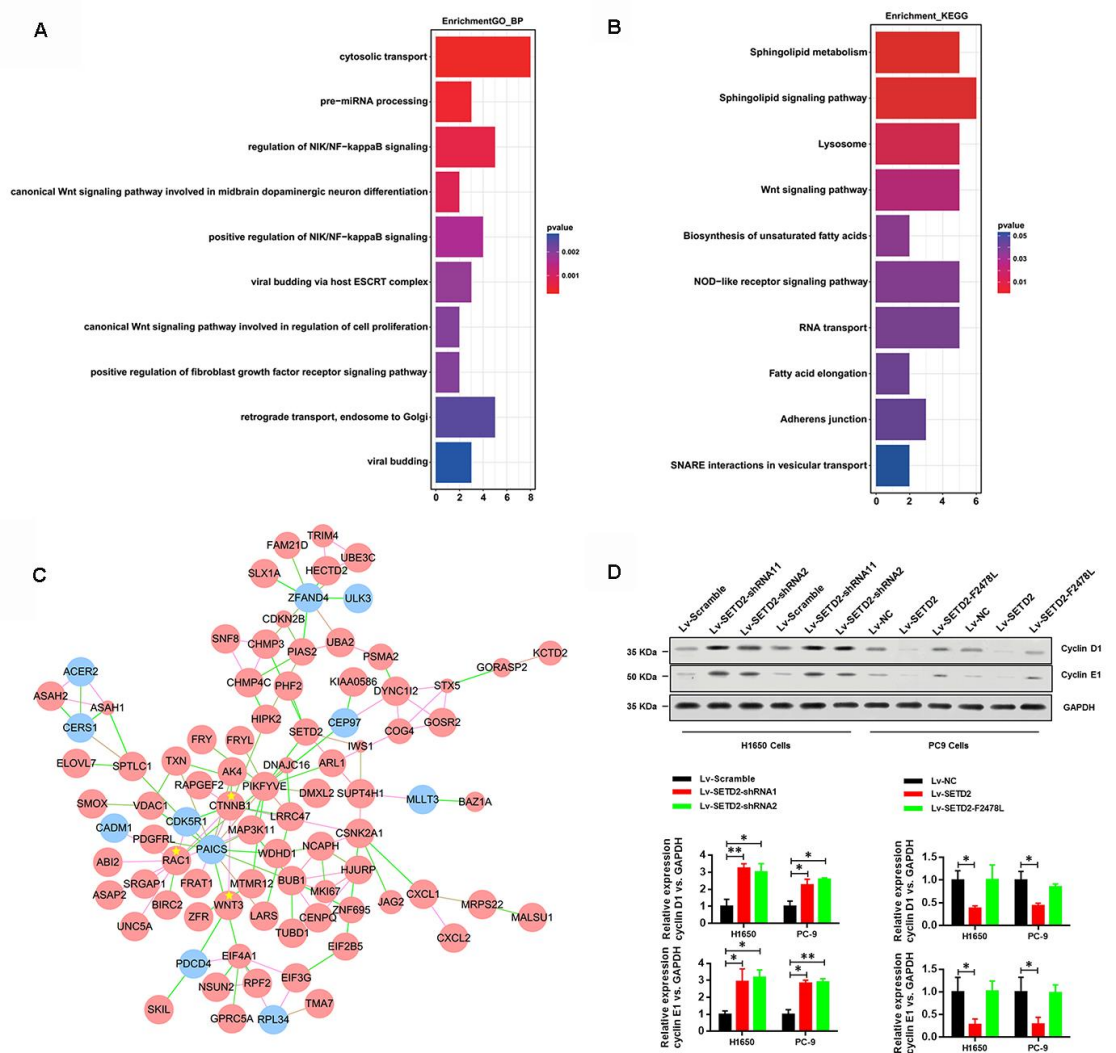
**SETD2 suppressed cell cycle progression.** (**A**) GO and (**B**) KEGG analysis showed the altered pathways in the absence of SETD2. (**C**) Gene co-interaction network identified crucial roles of CTNNB1, WNT3 and RAC1 in lung cancer which were marked by asterisks. Green lines represented the negative regulation between two genes, and red lines represented positive regulation. (**D**) Protein levels of G1 phase checkpoints Cyclin D1 and Cyclin E1 upon SETD2 overexpression or knockdown in H1650 and PC-9 cells. Quantitative results were shown on the right. *P<0.05. Two-tailed Student’s *t* test.

To illustrate the mechanisms by which SETD2 depletion regulated CXCL1 to aggravate LUAD progression, we conducted functional annotation of genes positively correlated with CXCL1. Both Gene ontology (GO) enrichment analysis (Figure 5A) and Kyoto Encyclopedia of Genes and Genomes (KEGG) pathway analysis (Figure 5B) indicated that “Wnt signaling pathway” was the prominently enriched gene set related to cancer progression and cell proliferation in the absence of SETD2. More importantly, gene co-expression network analysis emphasized the crucial roles of CTNNB1, WNT3 and RAC1 (Figure 5C). To further confirm the regulatory mechanism of SETD2 on cell cycle progression, we examined the expressions of G1 phase checkpoints and found that Cyclin D1 and Cyclin E1 were significantly up-regulated in SETD2-silenced H1650 and PC-9 cells (Figure 5D), whereas they were suppressed in wildtype SETD2-ovexpressing H1650 and PC-9 cells (Figure 5D). Therefore, these findings implicated that SETD2 depletion altered the cell cycle progression to accelerate LUAD progression.

### SETD2-catalyzed H3K36me3 interacts with CXCL1 promoter to regulate CXCL1 transcription

**Figure 6:**
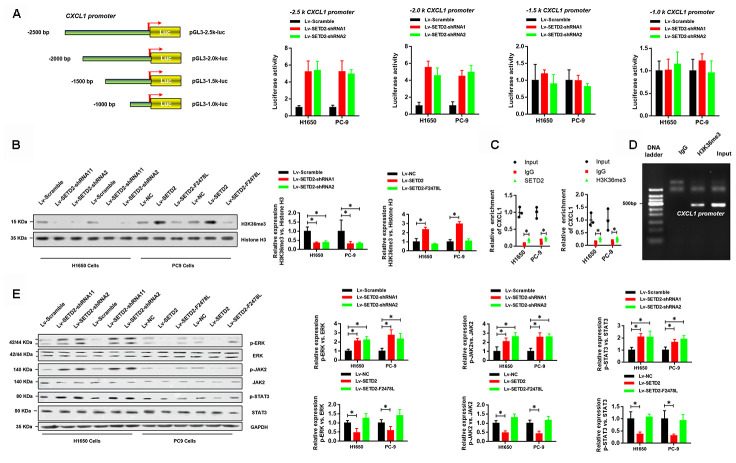
**SETD2-catalyzed H3K36me3 interacts with CXCL1 promoter to regulate CXCL1 transcription.** (**A**) A schematic diagram showing the CXCL1 promoter luciferase reporter vectors with four distinct promoter regions, which then were co-transfected with Lv-SETD2-shRNA1, Lv-SETD2-shRNA2, or Lv-Scramble into H1650 and PC-9 cells and subjected to luciferase activity assays. (**B**) Western blotting analyses of H3K36me3 levels upon SETD2 overexpression or depletion in H1650 and PC-9 cells. Quantitative results were indicated on the right panel. (**C**) ChIP-qPCR assays of SETD2 and H3K36me3 in CXCL1 gene. (**D**) ChIP assay of H3K36me3 in CXCL1 promoter. (**E**) Western blotting analyses of phosphorylated and total protein level of ERK, JAK2 and STAT3 upon SETD2 overexpression or depletion in H1650 and PC-9 cells. Quantitative results were indicated on the right panel. *P<0.05, **P<0.01. Two-tailed Student’s *t* test.

As aforementioned, SETD2 catalyzed the trimethylation of lysine 36 on histone 3 to regulate gene transcription and was implicated in RNA splicing during gene transcription. Therefore, we sought to answer how SETD2 regulates CXCL1 expression. Figure 6A shows that four truncations of CXCL1 promoter were amplified and cloned into pGL3-basic vectors for luciferase reporter assays. Results demonstrated that SETD2 ablation significantly increased the expressions of -2.5 k and -2.0 k CXCL1 promoter regions, while no changes were observed in the expressions of -1.5 k and -1.0 k CXCL1 promoter regions (Figure 6A), suggesting that SETD2 negatively regulated CXCL1 expression *via* its -2.0 k~-1.5 k promoter region.

Next, we examined whether SETD2-catalyzed H3K36me3 was associated with CXCL1 expression. Western blotting analyses were performed using cell lysates from H1650 and PC-9 cells with or without SETD2 overexpression or depletion. Results indicated that the H3K36me3 level was significantly increased or reduced upon SETD2 overexpression and ablation, respectively (Figure 6B). Moreover, independent ChIP-qPCR experiments in H1650 and PC-9 cells using antibodies against SETD2 and H3K36me3 showed that SETD2 and H3K36me3 were present at CXCL1 gene loci (Figure 6C). In addition, DNA gel electrophoresis results showed enrichment of CXCL1 promoter in anti-H3K36me3 ChIP assays (Figure 6D). Furthermore, SETD2 silence dramatically activated the signaling of ERK and JAK/STAT3 signaling pathways, which were indicated by the elevated level of phosphorylated ERK, JAK2 and STAT3 (Figure 6E). Taken together, these results revealed that SETD2-catalyzed H3K36me3 interacted with CXCL1 promoter to regulate its transcription and downstream signaling pathways.

### SETD2-CXCL1 axis inhibits the cell proliferation ability and cell cycle in LUAD cells *in vitro* and *in vivo*

**Figure 7:**
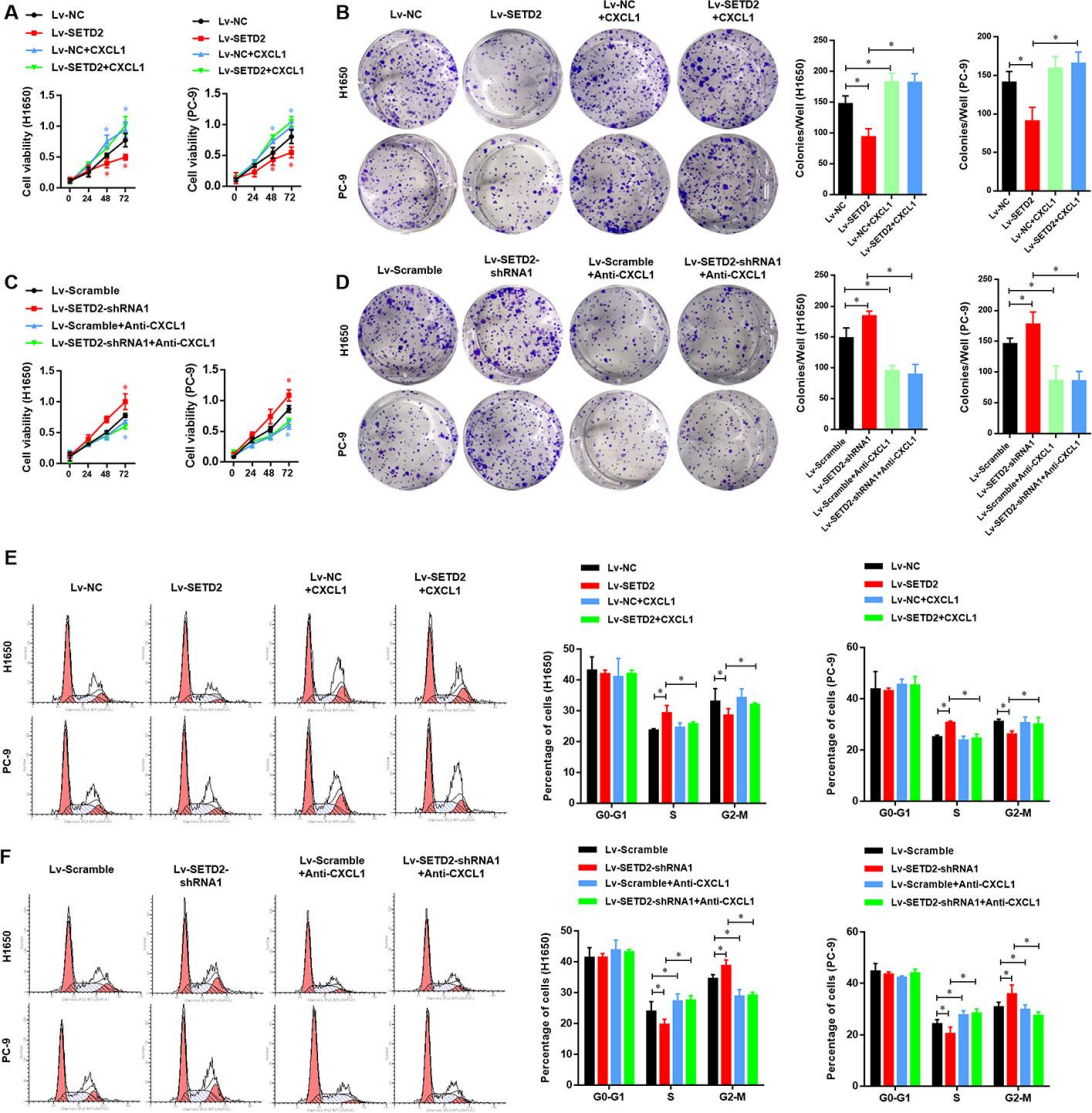
**SETD2-CXCL1 axis inhibits the cell proliferation ability and cell cycle in LUAD cells *in vitro*.** (**A**) Cell proliferation and colony formation assay (**B**) evaluated the effect of exogenous CXCL1 treatment in SETD2 overexpressed LUAD cells, compared with vector control LUADs cells. (**C**) Cell proliferation and colony formation assay (**D**) evaluated the effect of endogenous CXCL1 neutralization by Anti-CXCL1 antibody in SETD2 deficiency LUAD cells, compared with vector control LUADs cells. (**E**) Cell cycle analysis of the effect of exogenous CXCL1 treatment in SETD2 overexpressed LUAD cells, compared with vector control LUADs cells. (**F**) Cell cycle analysis of the effect of endogenous CXCL1 neutralization by Anti-CXCL1 antibody in SETD2 deficiency LUAD cells, compared with vector control LUADs cells. *P<0.05. One-way ANOVA with Dunn’s multiple-comparisons test.

We next evaluated the critical role of CXCL1 in the SETD2-overexpression or deficiency LUAD cells. First, we detected the effect of exogenous CXCL1 treatment in SETD2 overexpressed LUAD cells, compared with vector control LUAD cells. As shown in Figure 7A, 7B, the SETD2-impaired cell viability and colony formation ability of LUADs were obviously rescued by exogenous CXCL1 treatment. Similarly, the decreased G2-M phases caused by SETD2 overexpression eventually rose again, with the S phase relatively decreased (Figure 7E). On the contrary, endogenous CXCL1 neutralization by Anti-CXCL1 antibody significantly suppressed the cell viability and colony formation of LUAD cells (Figure 7C, 7D). Cell cycle analysis also suggested that endogenous CXCL1 neutralization reversed the enhanced G2-M phase and reduced S phase caused by SETD2 deficiency (Figure 7F).

**Figure 8:**
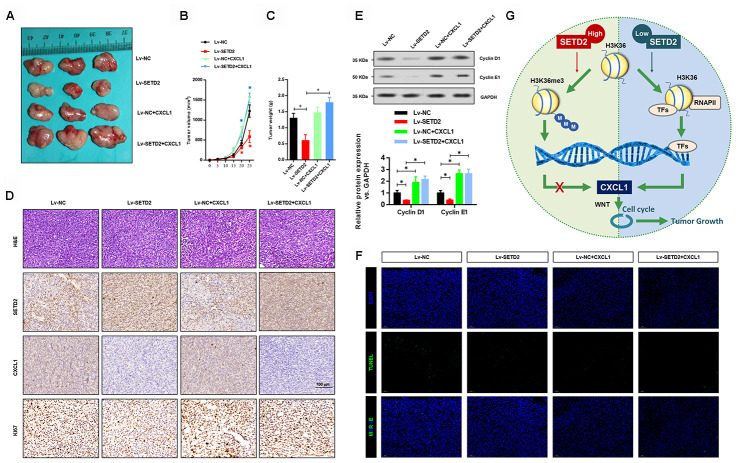
**SETD2 impairs lung cancer cell growth *in vivo*.** (**A**) A representative image of tumor volume of control and SETD2-overexpressing H1650 cells with or without CXCL1 overexpression. (**B**) Measurement of subcutaneous tumor growth of control and SETD2-overexpressing H1650 cells with or without CXCL1 overexpression. (**C**) Subcutaneous tumors were excised and weighed after mice were sacrificed. (**D**) H&E, SETD2 and CXCL1 staining of subcutaneous tumors of control and SETD2-overexpressing H1650 cells with or without CXCL1 overexpression. Scale bars: 50 μm. (**E**) Protein levels of G1 phase checkpoints Cyclin D1 and Cyclin E1 upon SETD2 or CXCL1 overexpression in H1650 cells generated xenograft tumors. (**F**) Apoptosis levels in the H1650 cells generated xenograft tumor were evaluated by TUNEL assay. (**G**) Graphic model of SETD2 functions in LUAD. SETD2-catalyzed H3K36me3 recruited specific transcription factors to negatively regulate CXCL1 transcription, in which activation of CXCL1 facilitated cell cycle progression, whereas inactivation of CXCL1 led to tumor growth suppression. *P<0.05, **P<0.01. One-way ANOVA with Dunn’s multiple-comparisons test.

We further validated the regulatory relationship between SETD2 and CXCL1 in nude mice xenograft model. SETD2 and CXCL1 were ectopically overexpressed separately or simultaneously in H1650 cells to establish the SETD2-overexpressing (Lv-SETD2), CXCL1-overexpressing (Lv-CXCL1) and SETD2/CXCL1 double overexpressing (Lv-SETD2+CXCL1) H1650 cells, which were subcutaneously injected to nude mice. Xenograft assays showed that SETD2 overexpression dramatically reduced tumor growth compared with the control group (Figure 8A), while CXCL1 overexpression reversed tumor volume (Figure 8B) and weight (Figure 8C) regardless of the SETD2 expression level. H&E staining and IHC staining confirmed the expression levels of SETD2 and CXCL1 in these tumors (Figure 8D). Furthermore, we confirmed the regulatory mechanism of SETD2 on cell cycle progression *via* evaluating the expressions of G1 phase checkpoints (Cyclin D1 and Cyclin E1). We found that Cyclin D1 and Cyclin E1 were significantly down-regulated in wildtype SETD2-overexpressed xenograft tumors (Figure 8E), whereas they were elevated after CXCL1 overexpressed (Figure 8E). Consistent with the *in vitro* data, the overexpression of SETD2 or CXCL1 showed no effect on the apoptosis of H1650 cells *in vivo*, as indicated by TUNEL assay (Figure 8F). These results suggested the modulatory effects of SETD2/CXCL1 axis on tumor growth in LUAD.

## DISCUSSION

Cancer cells acquire molecular changes in a different way from those in normal cells, potentially exposing them to new epigenetic vulnerabilities [18]. Therefore, important epigenetic regulators can be implicated as potential therapeutic targets. SETD2 and its catalyzed H3K36me3 play crucial roles in maintaining chromosome integrity and regulating gene transcription [19–22]. SETD2 is frequently mutated or deleted in spectrum of human cancers [23–28]. In the present study, we found that SETD2 was significantly decreased in LUAD. SETD2 depletion greatly up-regulated CXCL1 *via* its enzymatic activity in catalyzing H3K36me3, which stimulated Wnt-dependent cell cycle progression and promoted tumorigenesis (Figure 8E).

Previous studies using murine models have revealed that activation of Wnt signaling is associated with initiation and progression of lung cancer [29]. Several Wnt pathway molecules have been elevated in LUAD, such as WNT1, WNT2, WNT3 and β-catenin [30–32]. In line with these results, our RNA-Seq analysis revealed that Wnt signaling pathway and its related key molecules were implicated in tumorigenesis upon SETD2 depletion. Although our results indicated that SETD2 exerted its function in a manner largely dependent on canonical Wnt signaling, several other important signaling pathways (e.g. NF-κB signaling and cytosolic transport process) were also altered in the absence of SETD2. Therefore, other signaling pathways might also contribute to the impact of SETD2 on tumorigenesis.

Chemokines and their receptors play important roles in different biological processes, such as inflammation, immune responses and angiogenesis [33]. Penetration of inflammatory cells and chemokines into tumors is regarded as an important gist for clinical prognosis of cancer [34]. The chronic inflammation in many organs, like gastrointestinal, prostate, lung and bladder, can increase the risks of cancer and promote the cancer progression [35–39]. Chemokine CXCL1 is a monomeric protein of chemotaxis cytokines, which specifically binds to its receptor CXCR2 [40] and has been reported to promote tumor growth and metastasis in various cancers. There is no CXCL1 expression in normal melanin cells, while the sustained expression of CXCL1 promotes malignant transformation and tumor growth of melanoma *via* autocrine, as well as microvascular growth into tumors in paracrine fashion [41–44]. Additionally, CXCL1 is abnormally up-regulated in many other cancers, such as colorectal cancer, breast cancer, bladder cancer and epithelial ovarian cancer [45–48]. Our results were consistent with these previous studies, in which the CXCL1 expression was up-regulated by SETD2 depletion in LUAD. Moreover, the elevated CXCL1 levels affected cell cycle progression and promoted cancer cell proliferation and colony formation.

Our results indicated that SETD2 and its catalyzed H3K36me3 interacted with CXCL1 promoter to regulate the CXCL1 expression. It is widely reported that H3K36me3 facilitates transcription [49], while in this study, we initially showed that SETD2 mediated H3K36me3 suppressed the expression of CXCL1. Nevertheless, the transcription factors involved remained undetermined here. According to previous reports, several transcription factors have participated in the regulation of CXCL1 expression, such as Sp1, NF-κB and HMGI (Y), which interact with corresponding *cis*-acting elements to stimulate CXCL1 promoter activity [50]. In our GO analysis using differentially expressed genes between control and SETD2-silenced H1650 cells, “positive regulation of NF-κB signaling” was enriched, which could partially explain how SETD2 regulated CXCL1 transcription.

In summary, our findings highlighted the inhibitory effects of SETD2 in LUAD with potential implications for cancer intervention. Moreover, our results established a role for SETD2- or H3K36me3-mediated cell growth aberration in tumorigenesis and might provide guidance for further understanding the molecular mechanisms associated with SETD2 deficiency.

## MATERIALS AND METHODS

### TCGA dataset

The gene expression profile of LUAD patients was downloaded from the TCGA database (https://portal.gdc.cancer.gov/), including 513 tumor samples and 59 normal samples. The gene expression levels were quantified as FPKM (fragments per kilobase per million mapped reads) by normalizing the mRNA length and library size after the read counts were generated using TopHat and HTSeq-count. To enable the log2 transformation, the zero values of the expression data were replaced with the minimum nonzero FPKM values. The differential expression of the genes between the two groups was defined as *P* < 0.05.

### LUAD tissue specimens

A total of 20 paired human LUAD and adjacent normal epithelial tissues were acquired from the Third Affiliated Hospital of Soochow University between 2014 and 2017. Tissue specimens were obtained through surgey and stored in liquid nitrogen at -80° C. All human tissue-related experiments were approved by the Ethical Committee of the Third Affiliated Hospital of Soochow University. Written informed consents were obtained from all patients before surgery.

### Expression plasmids and siRNA

The full-length human SETD2 and CXCL1 cDNAs were cloned into pLVX-IRES-Puro vector (Clontech) to generate SETD2 and CXCL1 expression plasmids, respectively. The siRNA targeting SETD2 was purchased from GenePharma. Three independent shRNA expression plasmids targeting SETD2 were generated by cloning annealed shRNA oligonucleotides into the PLKO.1-TRC vector. The shRNA sequences were listed as follows: shSETD2-1 forward: 5′-CCGGAGTAGTGCTTCCCGTTATAAACTCGAGTTTATAACGGGAAGCACTACTTTTTTG-3′, reverse: 5′-AATTCAAAAAAGTAGTGCTTCCCGTTATAAACTCGAGTTTATAACGGGAAGCACTACT-3′; shSETD2-2 forward: 5′-CCGGACGAATTAAAGACCGCAATAACTCGAGTTATTGCGGTCTTTAATTCGTTTTTTG-3′. reverse: 5′-AATTCAAAAAACGAATTAAAGACCGCAATAACTCGAGTTATTGCGGTCTTTAATTCGT-3′; shSETD2-3 forward: 5′-CCGGTTCCGACGAGGGTCATCATATCTCGAGATATGATGACCCTCGTCGGAATTTTTG-3′; reverse: 5′-AATTCAAAAATTCCGACGAGGGTCATCATATCTCGAGATATGATGACCCTCGTCGGAA-3′.

### Cell lines and cell culture

All cells used in this study were obtained from the Chinese Cell Bank of the Chinese Academy of Sciences (Shanghai, China). Human bronchial epithelial (HBE) cells and human lung cancer cells A549, H1975, H1299, H1650 and PC-9 were maintained in RPMI-1640 culture medium supplemented with 10% fetal bovine serum (FBS) and 1% penicillin/streptomycin (P/S) solution. Lentivirus was used to establish individual stable cell lines. siRNA duplexes targeting SETD2 (100 nM) and corresponding negative control (NC) oligonucleotides were transfected into cells using Lipofectamine 3000 (Invitrogen) according to the manufacturer’s instructions.

Cell proliferation assay was performed using CellTiter 96® Non-Radioactive Cell Proliferation Assay (MTT) kit (Promega) according to the manufacturer’s instructions. Briefly, cells were seeded into a 96-well plate (100 μL/well) at a density of 1×10^4^ cells/mL and cultured at 37° C in an incubator containing 5% CO_2_. Each well was added with 10 μL MTS solution and then incubated at 37° C for 2 h. The spectrophotometric absorbance at 590 nm was determined for each sample. All the experiments were performed in triplicate.

For soft agar colony formation assays, cells were suspended in RPMI-1640 containing 0.35% low-melting agar (Invitrogen) and 10% FBS and seeded onto a coating of 0.8% low-melting agar in RPMI1640 containing 10% FBS. Plates were incubated at 37° C in a humidified atmosphere containing 5% CO_2_. Colonies were counted after 3 or 4 weeks of culture. Each experiment was conducted in triplicate.

### RNA extraction and real-time qPCR

Total RNA was extracted using TRIzol reagent according to the manufacturer’s instructions. Next, 1 μg of purified RNA was reversely transcribed into first-strand cDNA using Superscript II (Invitrogen). Syber Green Universal Master Mix reagent (Roche) and primer mixtures were used for the real-time qPCR. GAPDH was used as the housekeeping gene. The primer sequences used for real-time qPCR were as follows: SETD2 forward: 5′-ATCGAGAGAGGACGCGCTATT-3′, reverse: 5′-AGGTACGCCTTGAGTATGTCTT-3′; CXCL1 forward: 5′-AGCTTGCCTCAATCCTGCATCC-3′; reverse: 5′-TCCTTCAGGAACAGCCACCAGT-3′.

### Western blotting analysis

Total proteins were extracted and subjected to SDS-PAGE. SETD2 antibody (Invitrogen), Cyclin D1 antibody (Cell Signaling Technology), Cyclin E1 antibody (Abcam), and H3K36me3 antibody (Cell Signaling Technology), phos-ERK antibody (Santa Cruz Biotechnology), ERK (Santa Cruz Biotechnology), phos-JAK2 (Santa Cruz Biotechnology), JAK2 (Santa Cruz Biotechnology), phos-STAT3 (Abcam) and STAT3 (Abcam) were used at a dilution of 1:2,000. H3 (Cell Signaling Technology, 1:2,000) and GAPDH (Santa Cruz Biotechnology, 1:2,000) were used as loading controls.

### Cell cycle analysis

Cells were digested by 0.25% trypsin when they were in their logarithmic growth phase, followed by centrifugation at 1,000 rpm for 5 min to obtain the cell pellet. After fixed in pre-cooled 70% ethanol at –20° C overnight, propidium iodide (PI) was added into cells. Cell cycle phases were examined by flow cytometry (Beckman). Each experiment was conducted in triplicate.

### Reporter vector construction and luciferase reporter assays

Four CXCL1 promoter truncations containing respective -2.5 k, -2.0 k, -1.5 k and -1.0 k regions of CXCL1 promoter were amplified and cloned into pGL3-basic vectors (Promega) to conduct specific CXCL1 promoter-luciferase reporter vectors.

The siNC or siSETD2 was co-transfected with distinct CXCL1 promoter reporter vector into H1650 and PC-9 cells using Lipofectamine 3000 (Invitrogen). The luciferase activity was determined at 48 h after transfection using the Dual-Luciferase Reporter Assay System (Promega) according to the manufacturer’s instructions. Each experiment was performed in triplicate.

### RNA-seq and data analysis

Total RNA from H1650 cells with or without SETD2 depletion was subjected to HiSeq RNA-Seq. Transcriptome reads from RNA-Seq experiments were mapped to the reference genome (hg19) using Hisat2 software. The gene expression level was quantified by the Ballgown package. *P* < 0.05 was considered statistically significant. The differentially expressed genes were subsequently analyzed for the enrichment of biological pathways using the ClusterProfiler package. Gene interaction network analysis was performed using STRING website (https://string-db.org/) and Cytoscape software (3.6.0). The RNA-seq data is available from the Gene Expression Omnibus database (https://www.ncbi.nlm.nih.gov/geo/) under accession number GSE150809.

### Chromatin immunoprecipitation (ChIP) assays

The ChIP assays were performed using the Magnetic ChIP kit (Millipore) according to the manufacturer’s instructions. Briefly, H1650 and PC-9 cells were fixed by 1% formaldehyde, fragmented by a combination of MNase and sonication. SETD2 (Active Motif) and H3K36me3 (Active Motif) antibodies were then used for immunoprecipitation. After washing and reverse-crosslinking, the precipitated DNA was amplified by CXCL1 promoter primers and then quantified by the Step-One Plus Real-Time PCR System and DNA gel electrophoresis.

### Immunohistochemistry (IHC) assays

Tumors from xenograft models were collected and then embedded by paraffin after fixed by 4% PFA. IHC analyses were performed using specific anti-SETD2 (Invitrogen) and anti-CXCL1 (Abcam) antibodies.

### Tumor xenografts

BALB/c nude mice (5 weeks old) were purchased from SLAC Animal Center (Shanghai, China) and then used for xenograft tumor model. The animal-related experiments were approved by the Institutional Animal Care and Use Committee of the Third Affiliated Hospital of Soochow University. H1650 cells were subcutaneously injected to nude mice, and then tumor volumes were monitored every 5 days. Tumor volumes were estimated by length and width and calculated using the following formula:

Tumor volume = (length * width^^2^)/2

About 1 month later, the nude mice were sacrificed, and then tumors were excised, pictured, and weighed.

### Statistical analysis

All experiments were performed in triplicate. GraphPad Prism 8.0 was used for statistical analyses. Data in all figures were presented as the mean ± SEM. Pearson correlation coefficients were used to evaluate the relationship between the expressions of SETD2 and CXCL1. Cox proportional hazards regression model and multivariate Cox proportional hazards model analyses were performed with the statistical software SPSS 22.0. Statistical significance was determined by multiple *t*-test, one-way ANOVA, two-way ANOVA, Pearson correlation coefficients or log-rank test. For computing gene signature scores based on expression profiling data from H1650 cells, genes were first z-normalized to the SD from the median across the cell samples, and the average of the z-normalized values for all the genes in the signature was used to represent the signature score for each sample profile. Survival rates were calculated using the Kaplan-Meier method and differences between survival curves were examined using a log-rank test. For all statistical tests, the 0.05 level of confidence (two-sided) was accepted for statistical significance.

### Ethics approval and consent to participate

All procedures followed were in accordance with the ethical standards of the Third Affiliated Hospital of Soochow University and obtained written informed consents from all the participants.

## Supplementary Material

Supplementary Figures

Supplementary Table 1
