# Supplementary material for: Histone methyltransferase SETD2 inhibits tumor growth via suppressing CXCL1-mediated activation of cell cycle in lung adenocarcinoma
Source: Aging (Albany NY). 2020 Nov 20;12(24):25189–206. doi: 10.18632/aging.104120 (PMC7803529; doi:10.18632/aging.104120)
Supplement: Supplementary Figures [file aging-12-104120-s001.pdf]

SUPPLEMENTARY FIGURES

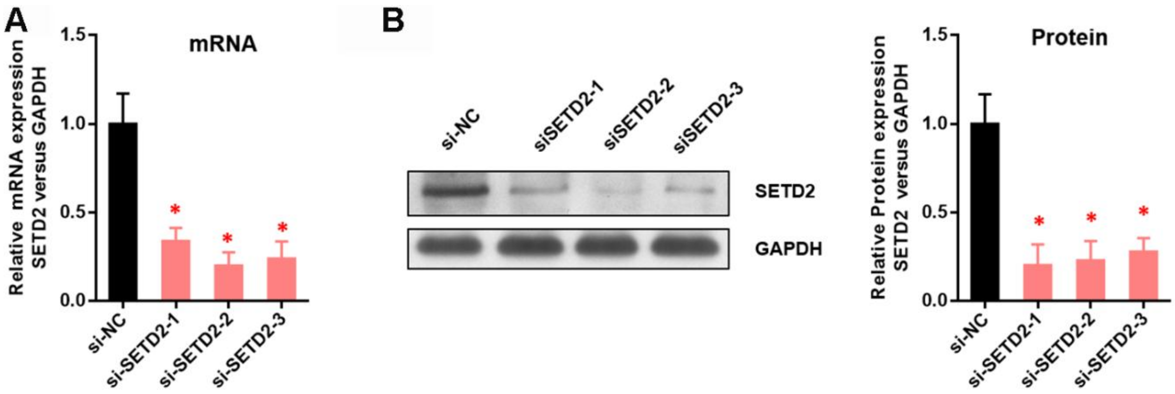

Supplementary Figure 1. The depletion efficiency of SETD2 in H1650 cells. (**A**) Real-time qPCR analysis of SETD2 expression at the mRNA level upon SETD2 siRNA transfection in H1650 cells. (**B**) Western blotting analysis of SETD2 expression at the protein level upon SETD2 siRNA transfection in H1650 cells.**P*< 0.05.

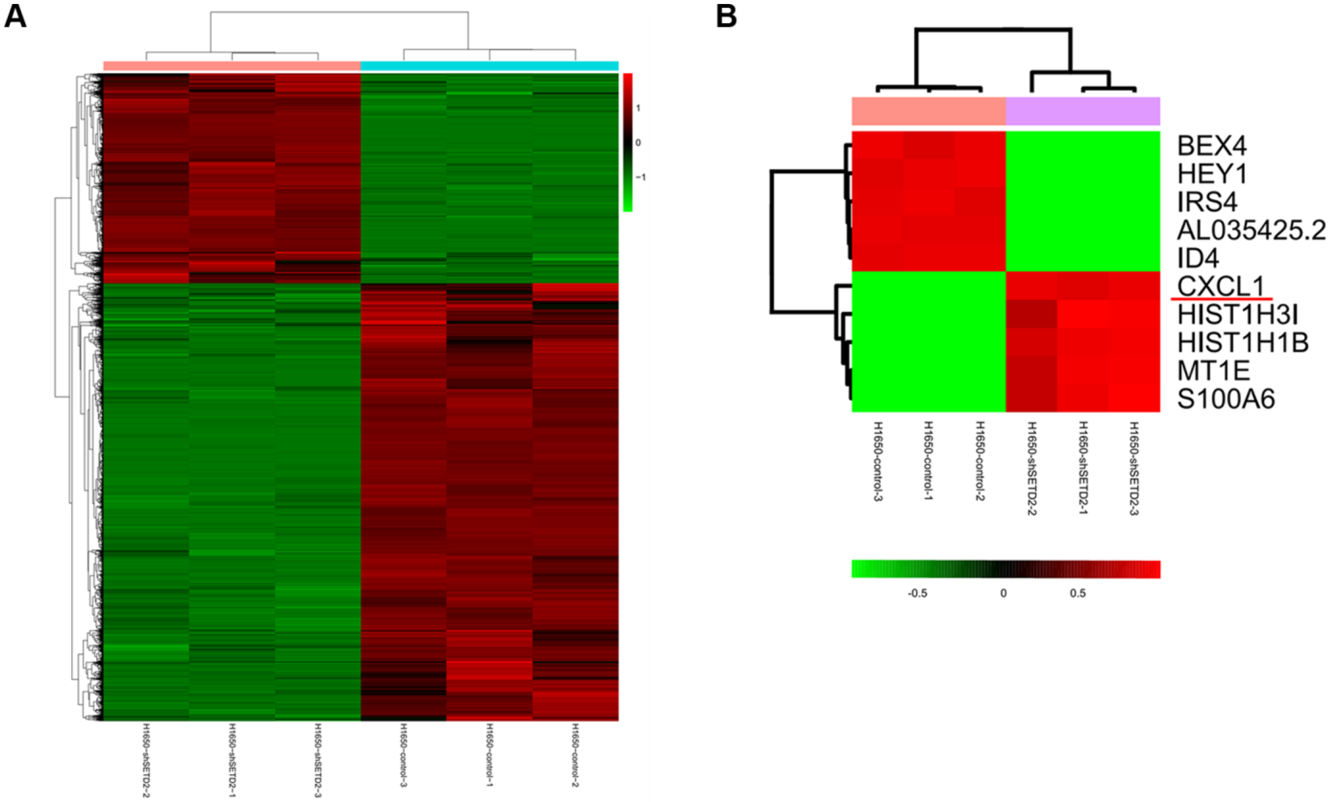

Supplementary Figure 2. CXCL1 is up-regulated upon SETD2 depletion. (**A**) Heat map indicating the differentially expressed genes in SETD2-silenced H1650 cells. (**B**) Heat map of top 10 differentially expressed genes in which CXCL1 was underlined by red.
